# Supplementary figures and images for: Moving analytical ultracentrifugation software to a good manufacturing practices (GMP) environment
Source: PLoS Comput Biol. 2020 Jun 19;16(6):e1007942. doi: 10.1371/journal.pcbi.1007942 (PMC7347214; doi:10.1371/journal.pcbi.1007942)

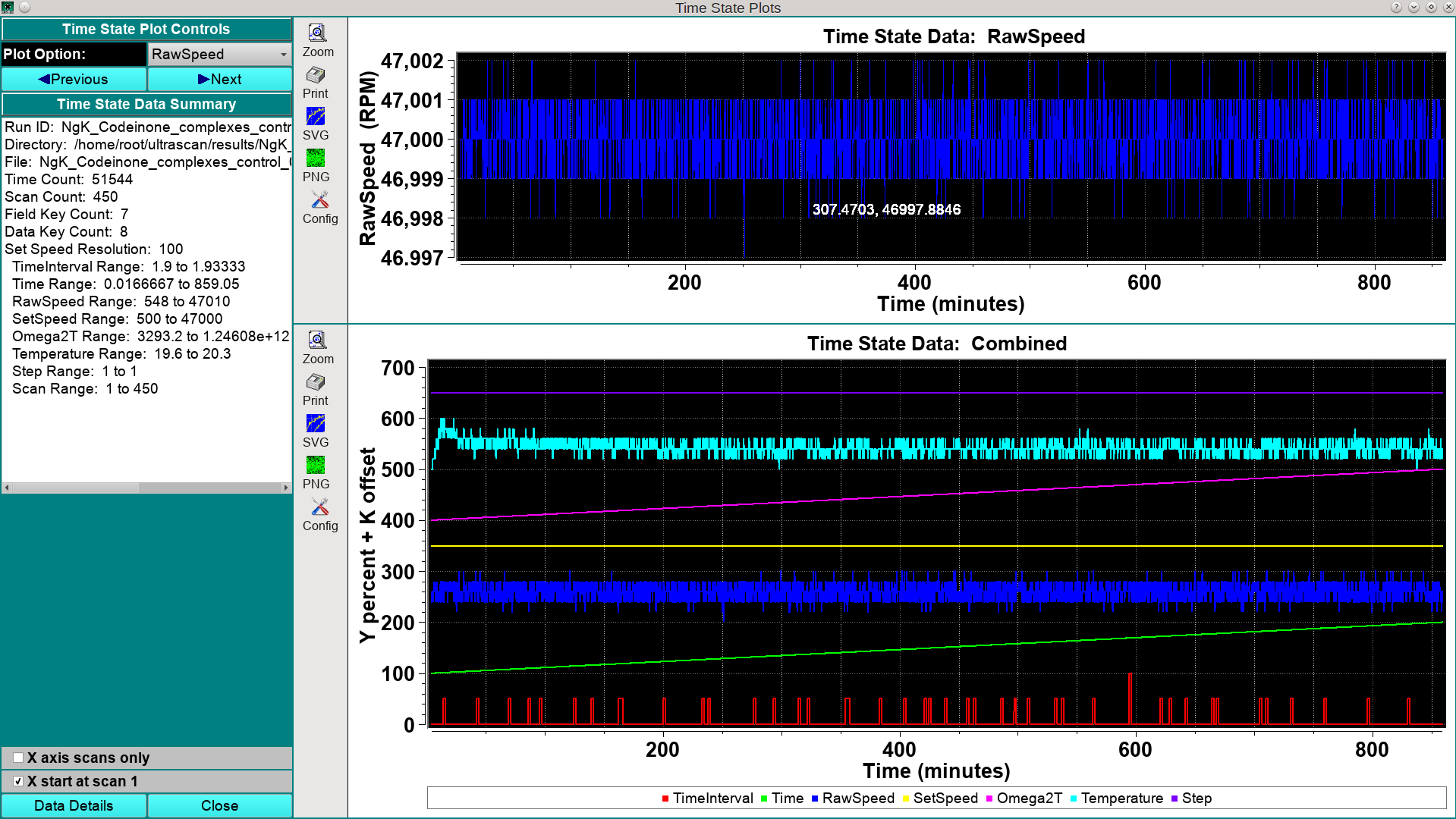

Supplement: S1 Fig — Actual temperature, rotor speed, scan interval, centrifugal force integral, time, set speed, and run stage are recorded in 1-second intervals and used to verify values recorded in scans (TIF) [file pcbi.1007942.s001.tif]

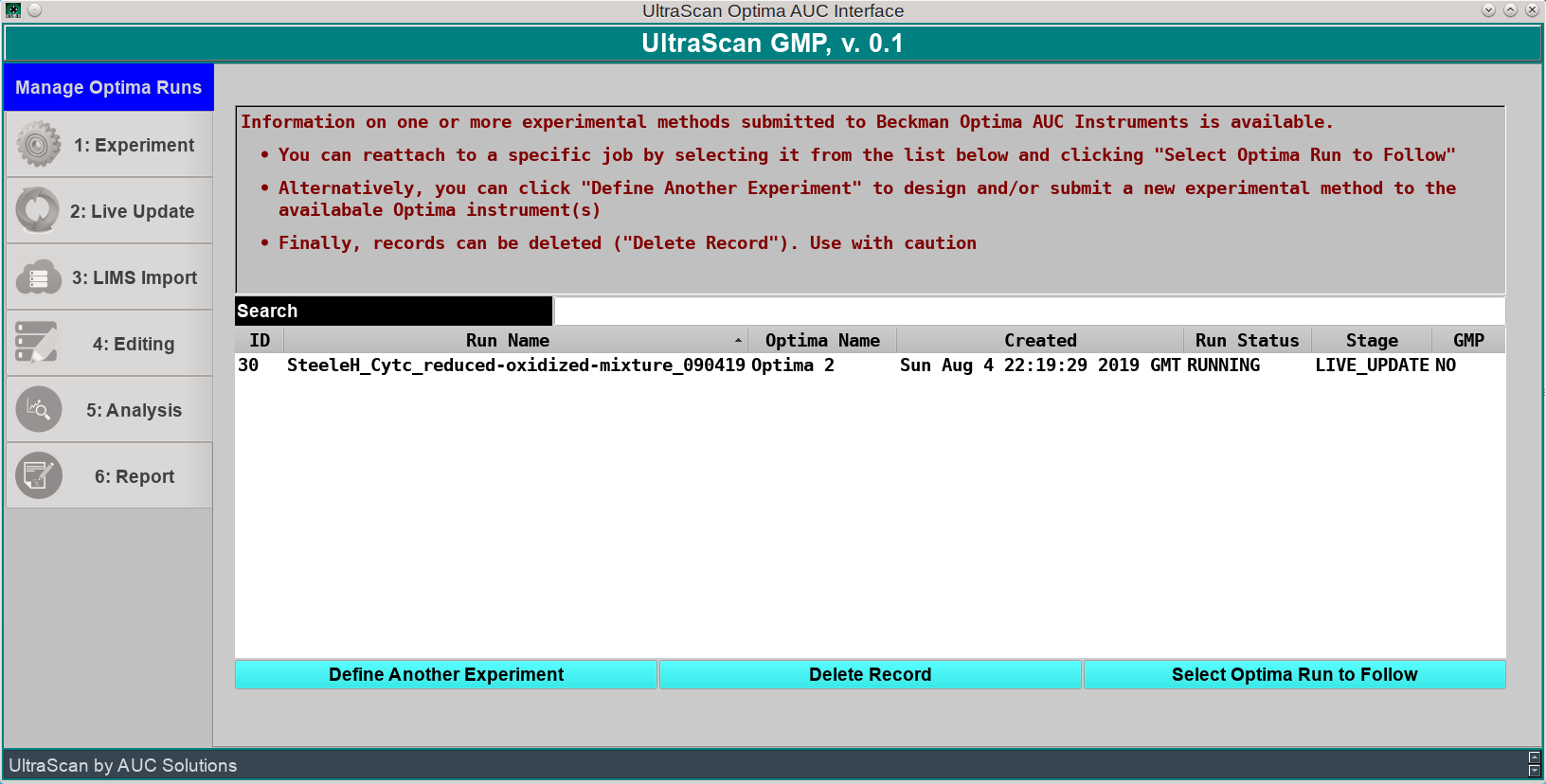

Supplement: S2 Fig — The user can re-attach to this experiment, or decide to design or load a profile for a different instrument that is currently idle. (TIF) [file pcbi.1007942.s002.tif]

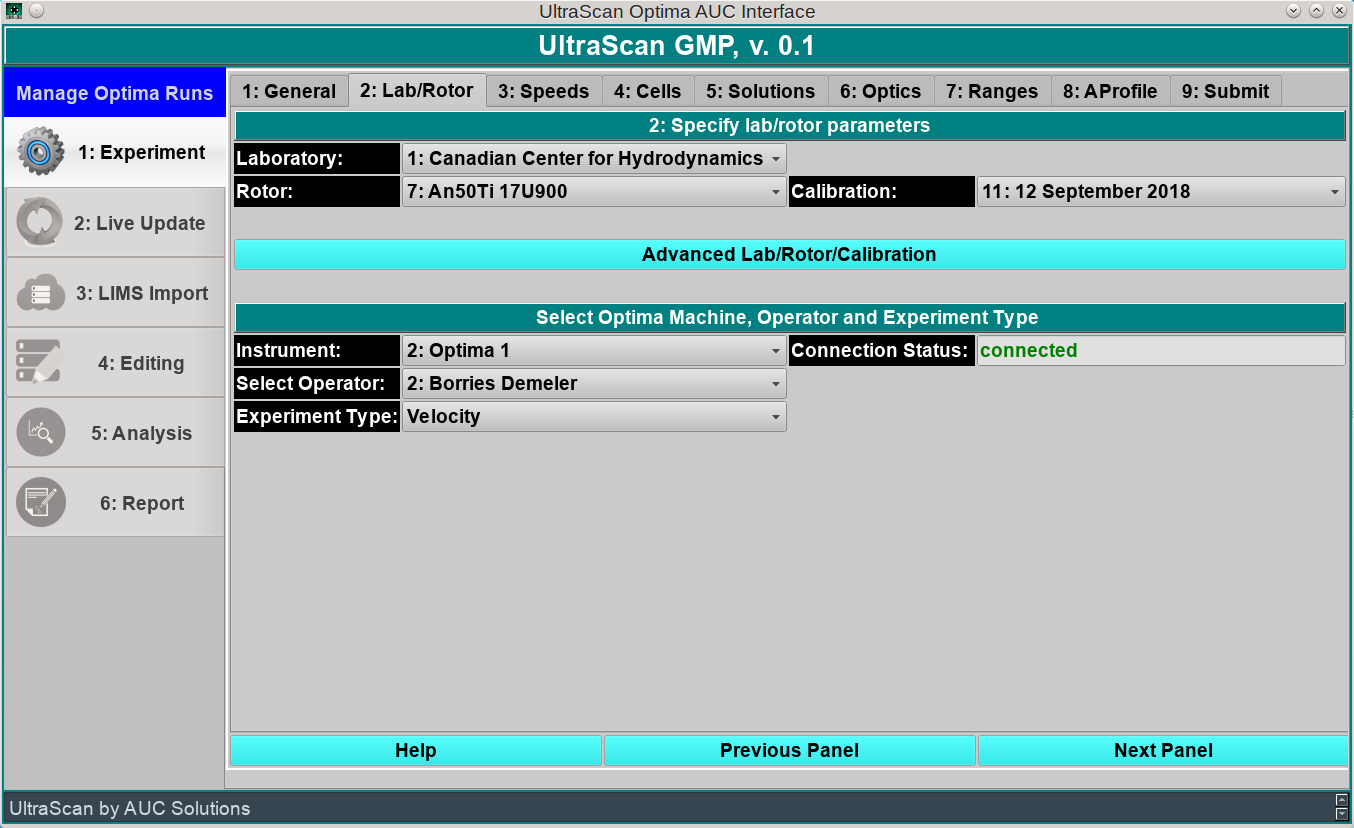

Supplement: S3 Fig — Also, the instrument, the operator performing the experiment is selected, as well as the experiment type. The instrument's connection status is checked by the program automatically. (TIF) [file pcbi.1007942.s003.tif]

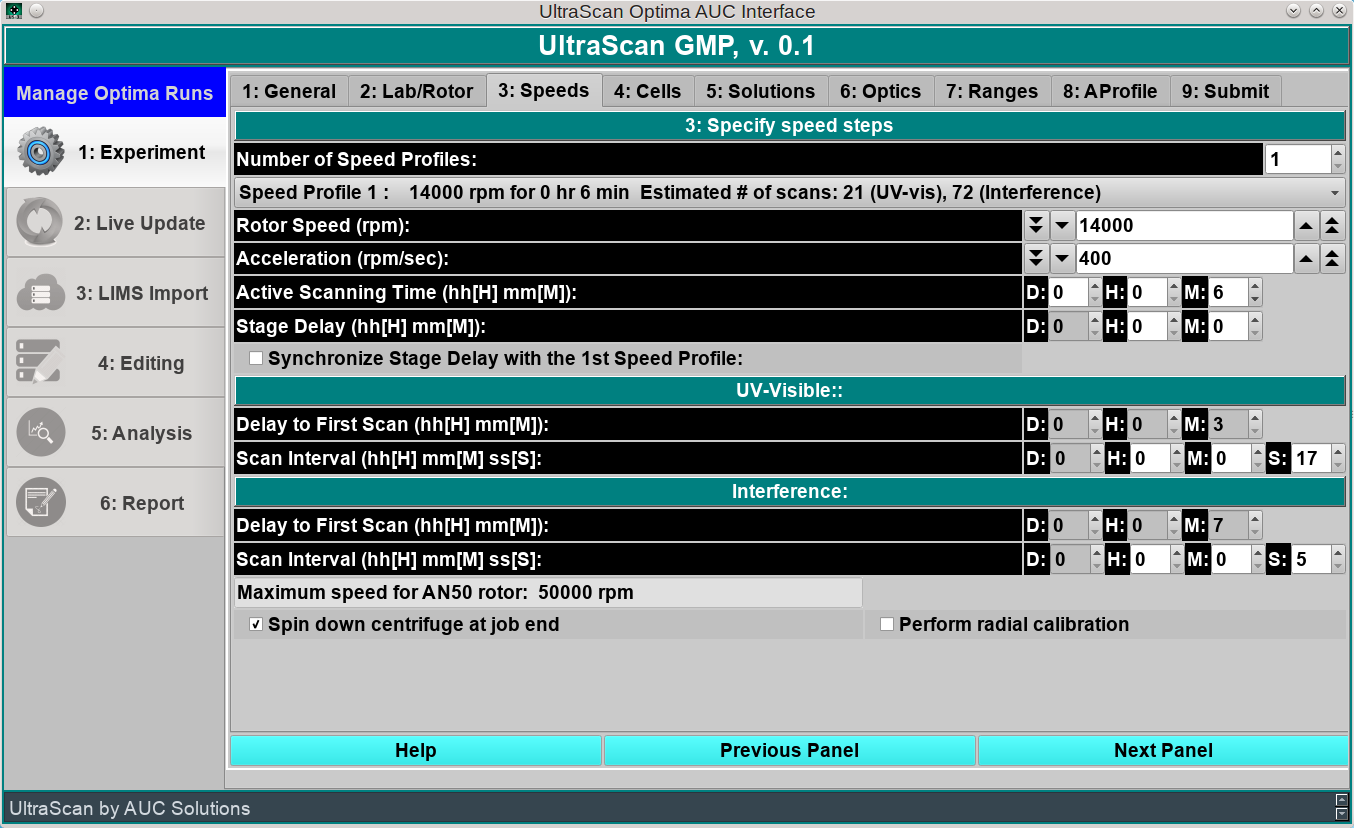

Supplement: S4 Fig — Minimum scan intervals are dependent on speed and calculated by the software automatically. Interference and UV/visible absorbance optics have different scan intervals and initial minimum delay times for the first scan that are instrument and speed dependent. (TIF) [file pcbi.1007942.s004.tif]

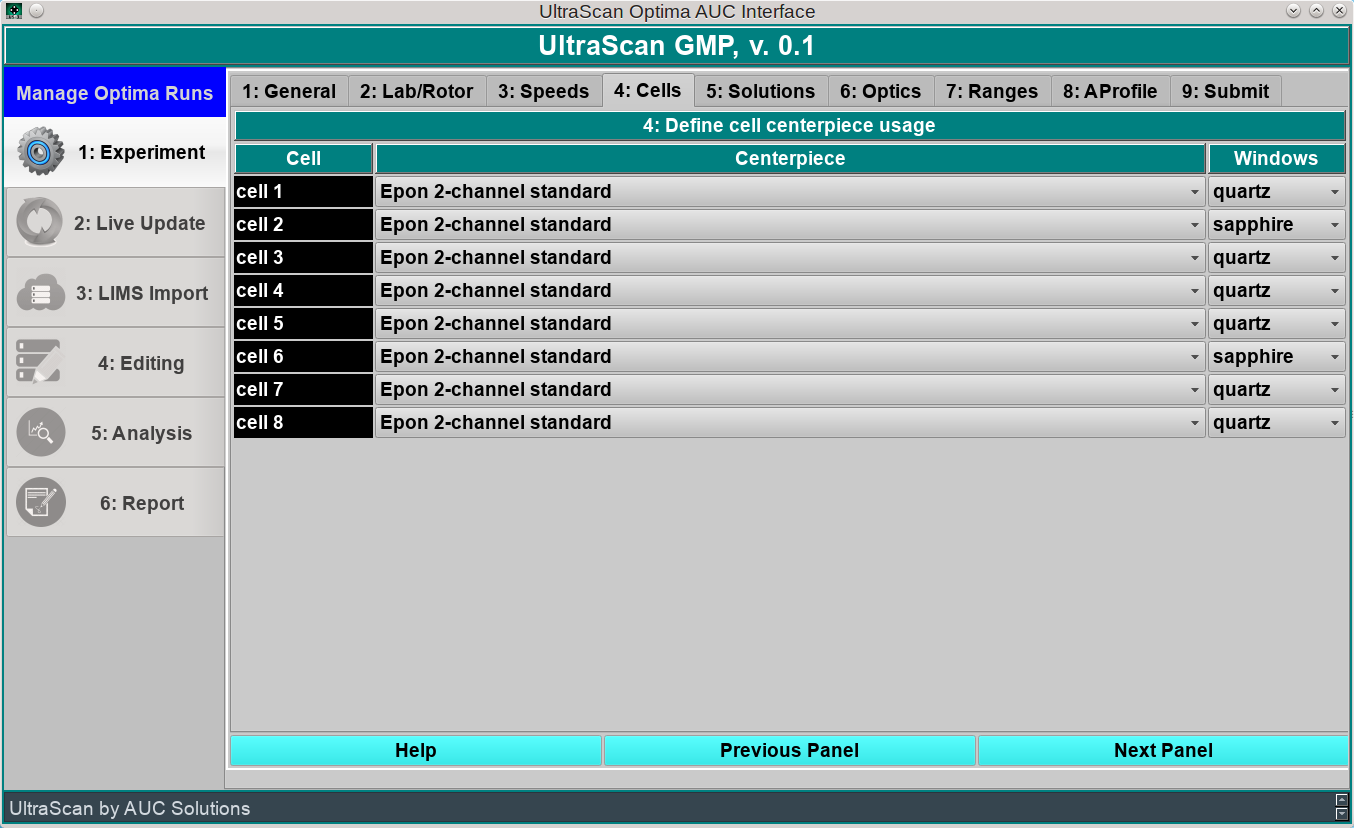

Supplement: S5 Fig — UltraScan is able to use all rotor positions for data acquisition and does not need to have a counterbalance located in hole 4 or 8, as long as a 3000 rpm radial calibration has already been performed with the counterbalance in place in a previous calibration run. For GMP experiments, the calibration centerpiece can be included instead to validate radial calibration. (TIF) [file pcbi.1007942.s005.tif]

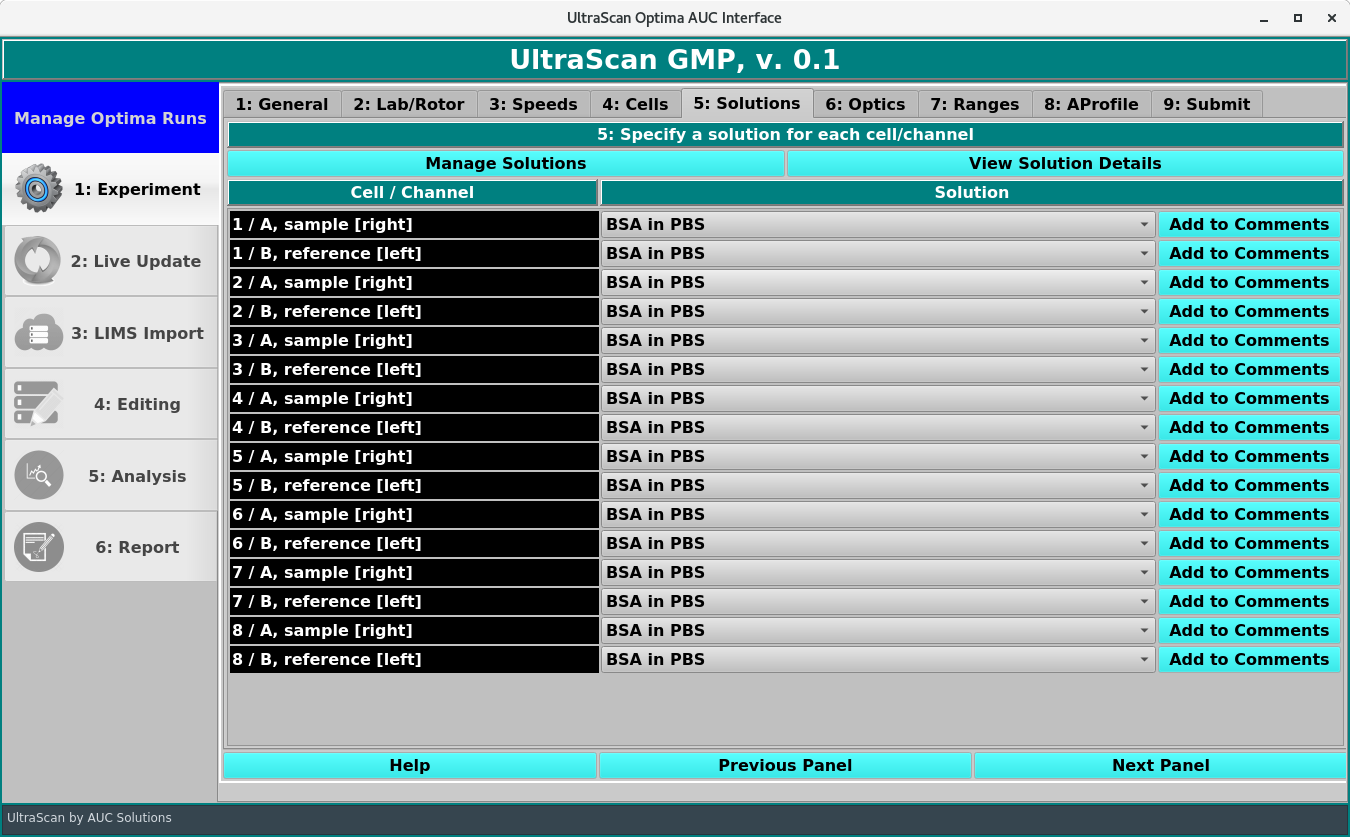

Supplement: S6 Fig — Analytes provide a partial specific volume and extinction coefficient to the analysis, buffer composition is needed to estimate density and viscosity for corrections to standard conditions. Additional solution comments can be added, new solutions can be defined. (TIF) [file pcbi.1007942.s006.tif]

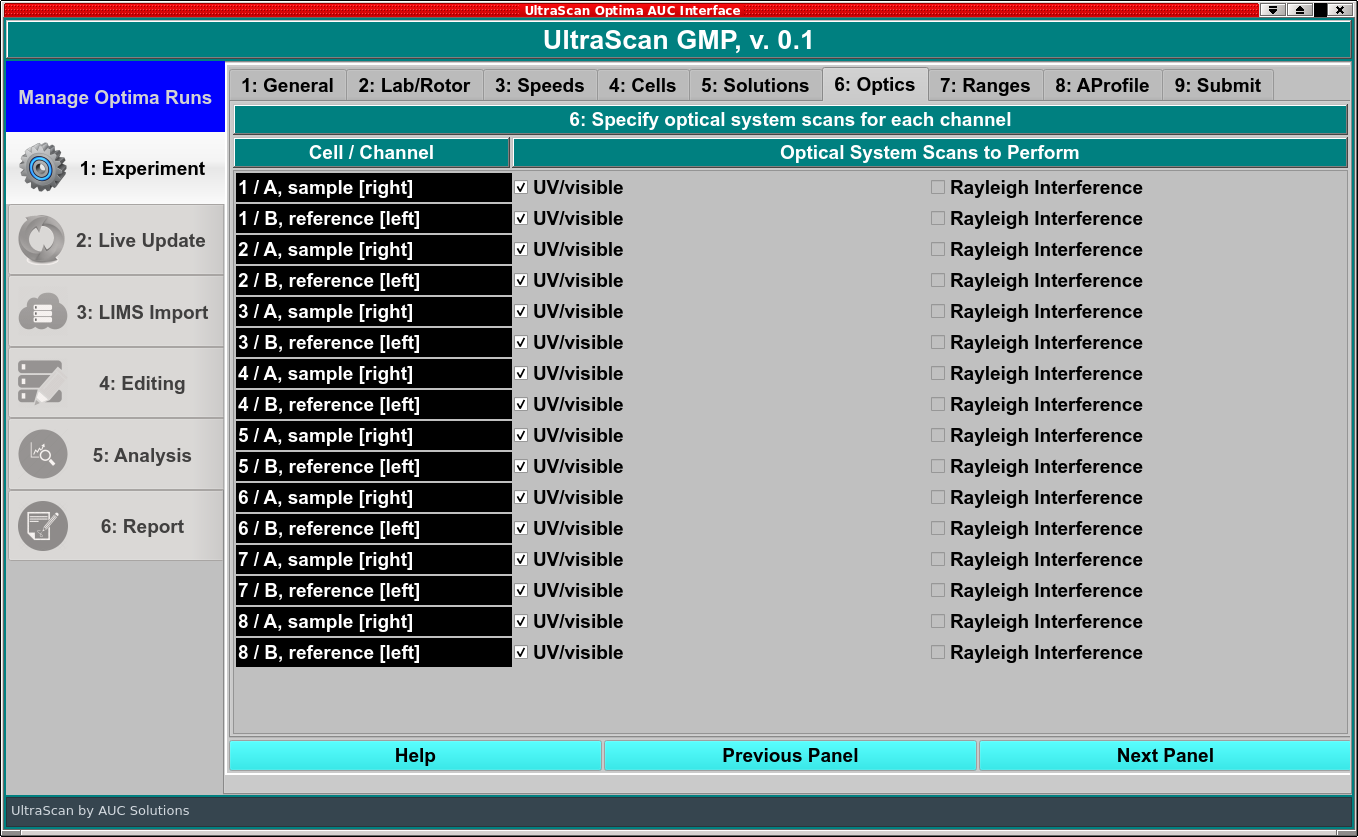

Supplement: S7 Fig — (TIF) [file pcbi.1007942.s007.tif]

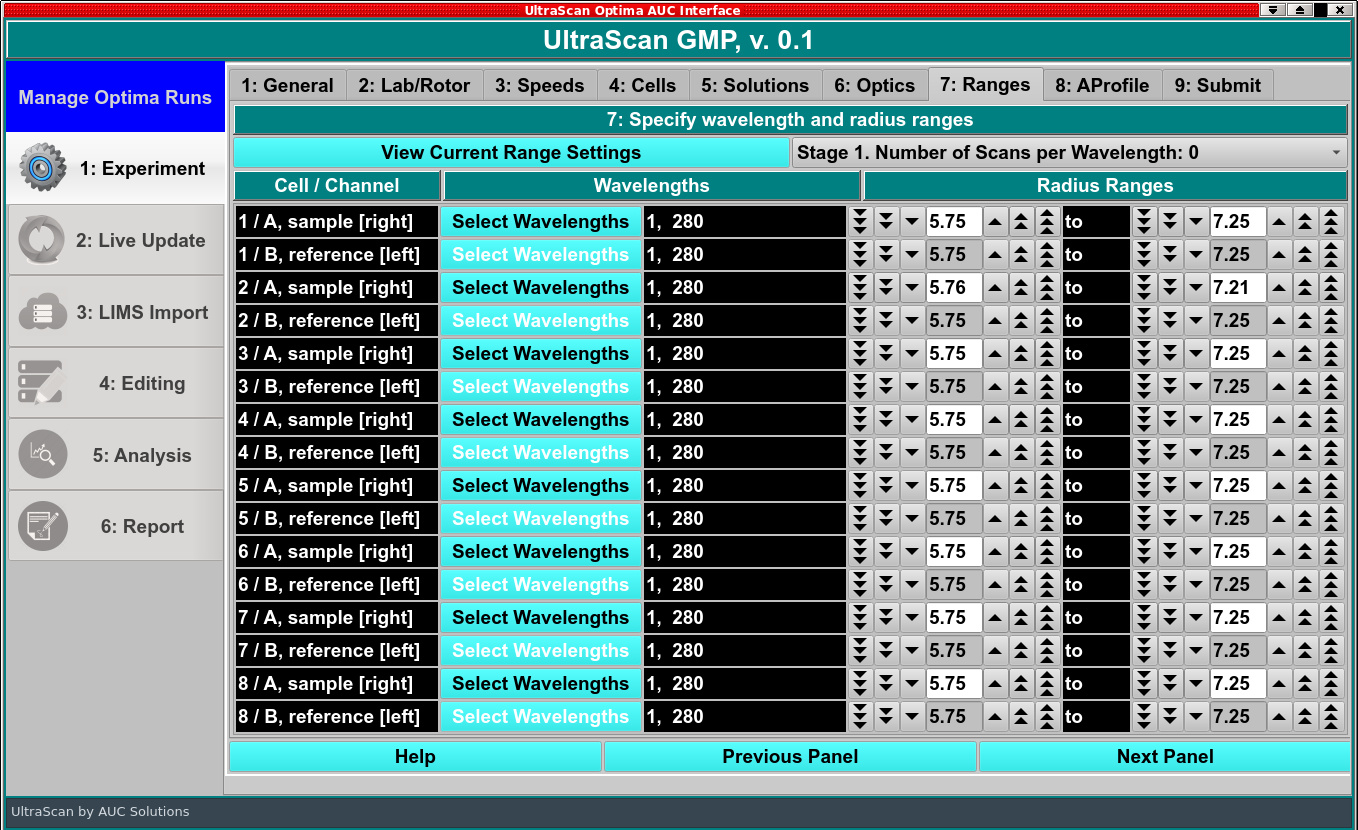

Supplement: S8 Fig — (TIF) [file pcbi.1007942.s008.tif]

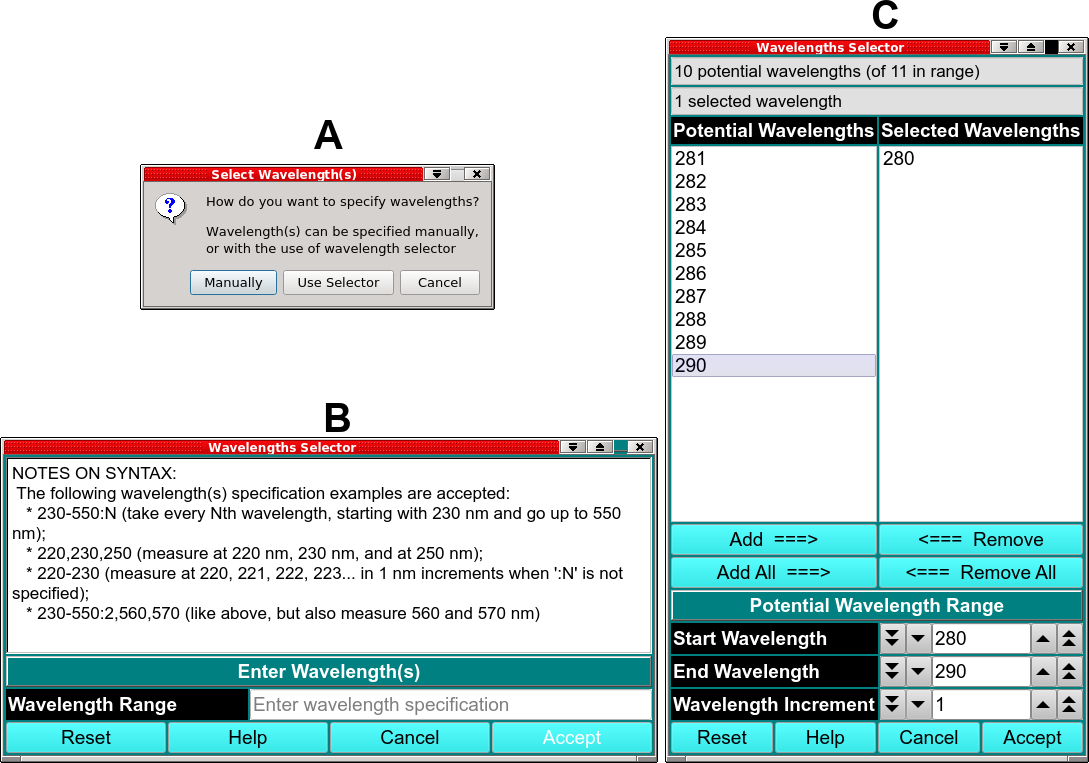

Supplement: S9 Fig — Wavelength selection: The user is prompted with a dialog (A) to choose between manual definition (B) or a selector dialog (C) to define wavelength ranges. The manual selector provides examples for a simple scripting language to define a series of wavelength choices. In the wavelength selector desired wavelength are selected by clicking on them in a GUI. (TIF) [file pcbi.1007942.s009.tif]

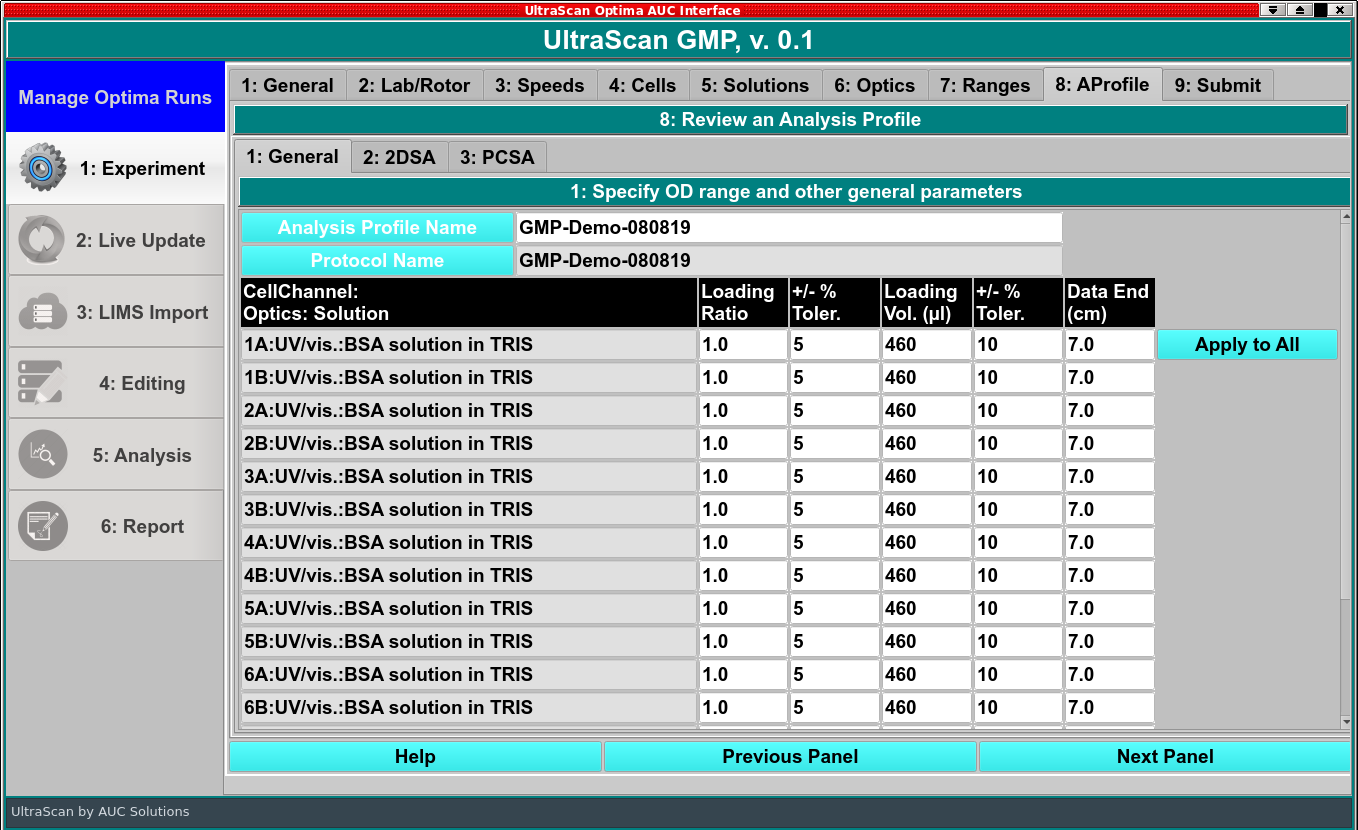

Supplement: S10 Fig — Concentration ratios and loading volumes can be associated with a tolerance limit for the reporting section. For replicate samples an “Apply to All” button replicates settings of the first sample to the other rows. (TIF) [file pcbi.1007942.s010.tif]

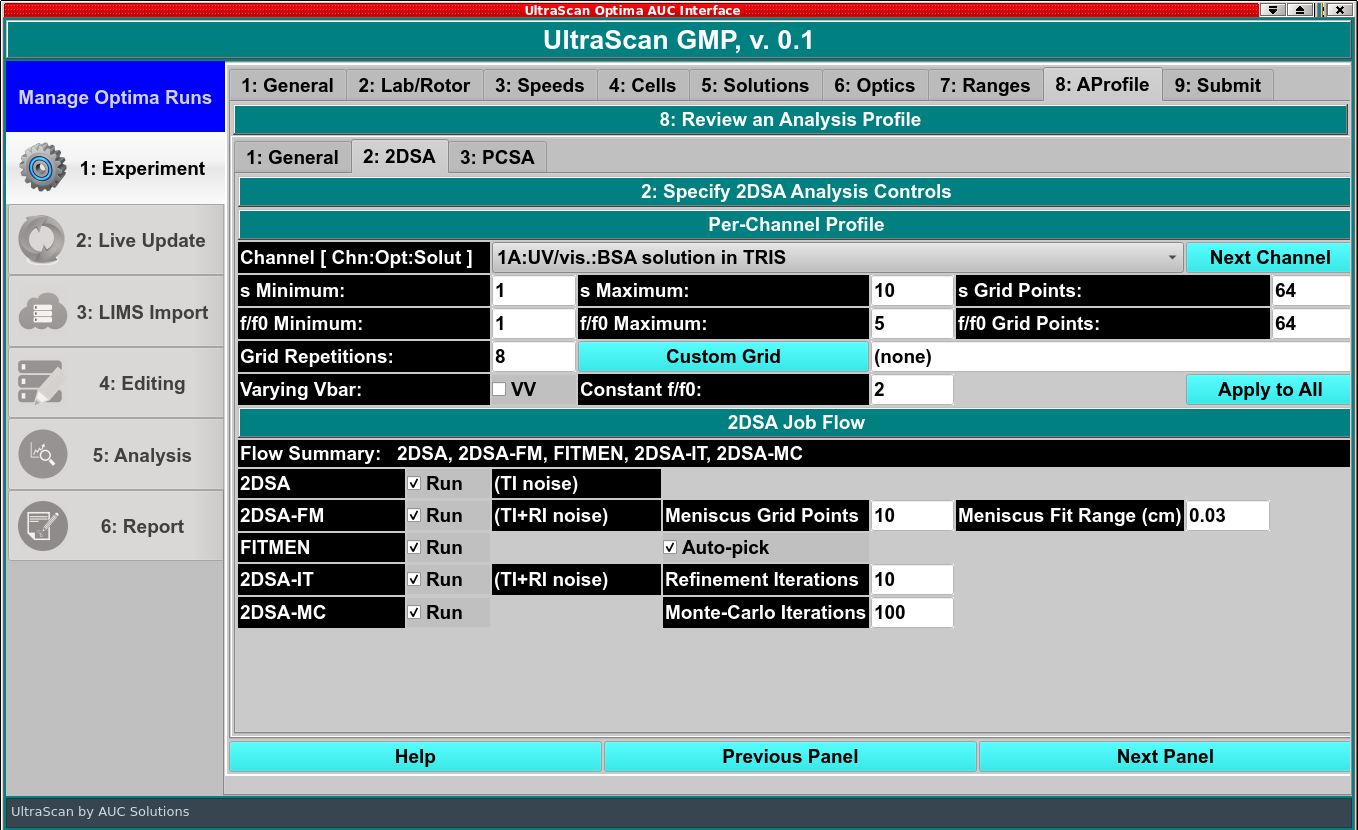

Supplement: S11 Fig — (TIF) [file pcbi.1007942.s011.tif]

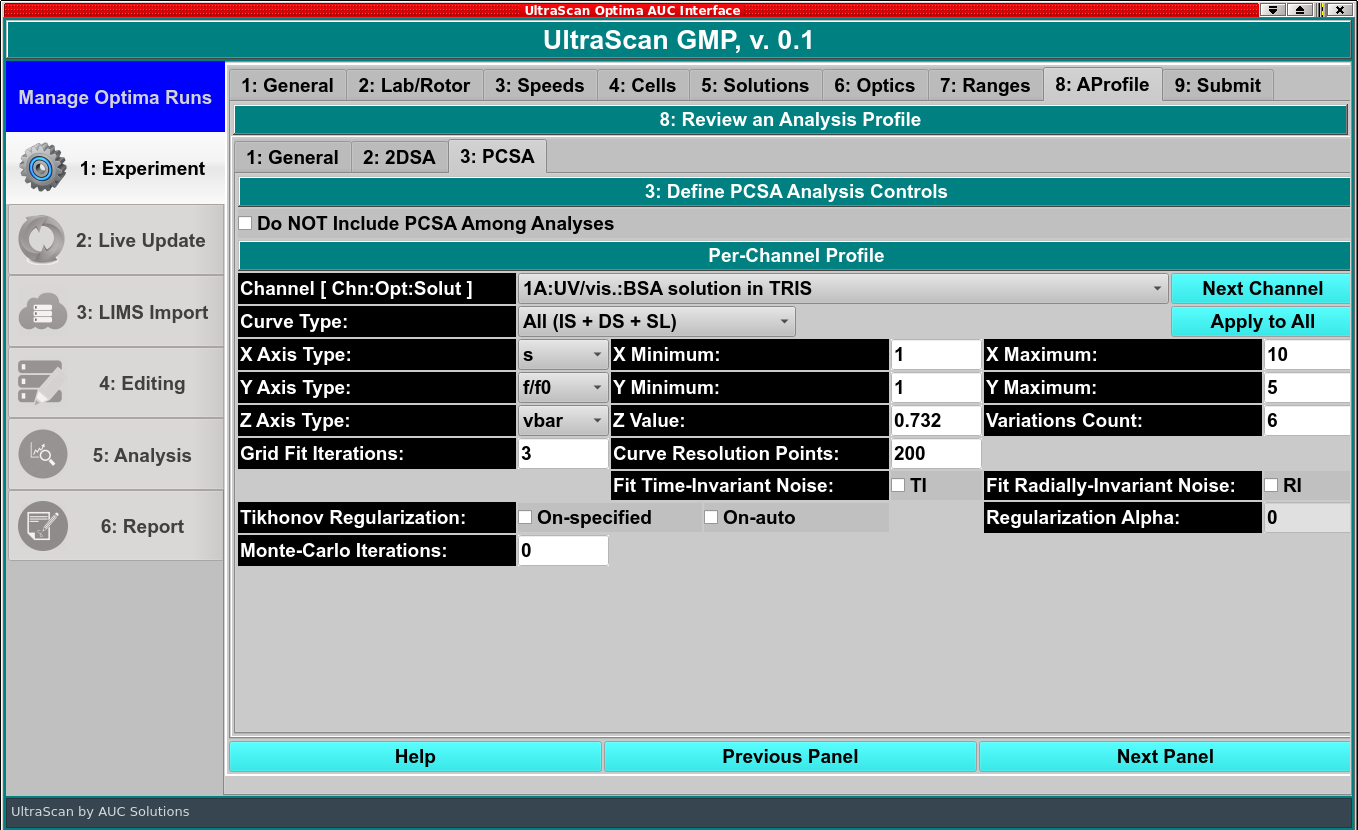

Supplement: S12 Fig — (TIF) [file pcbi.1007942.s012.tif]

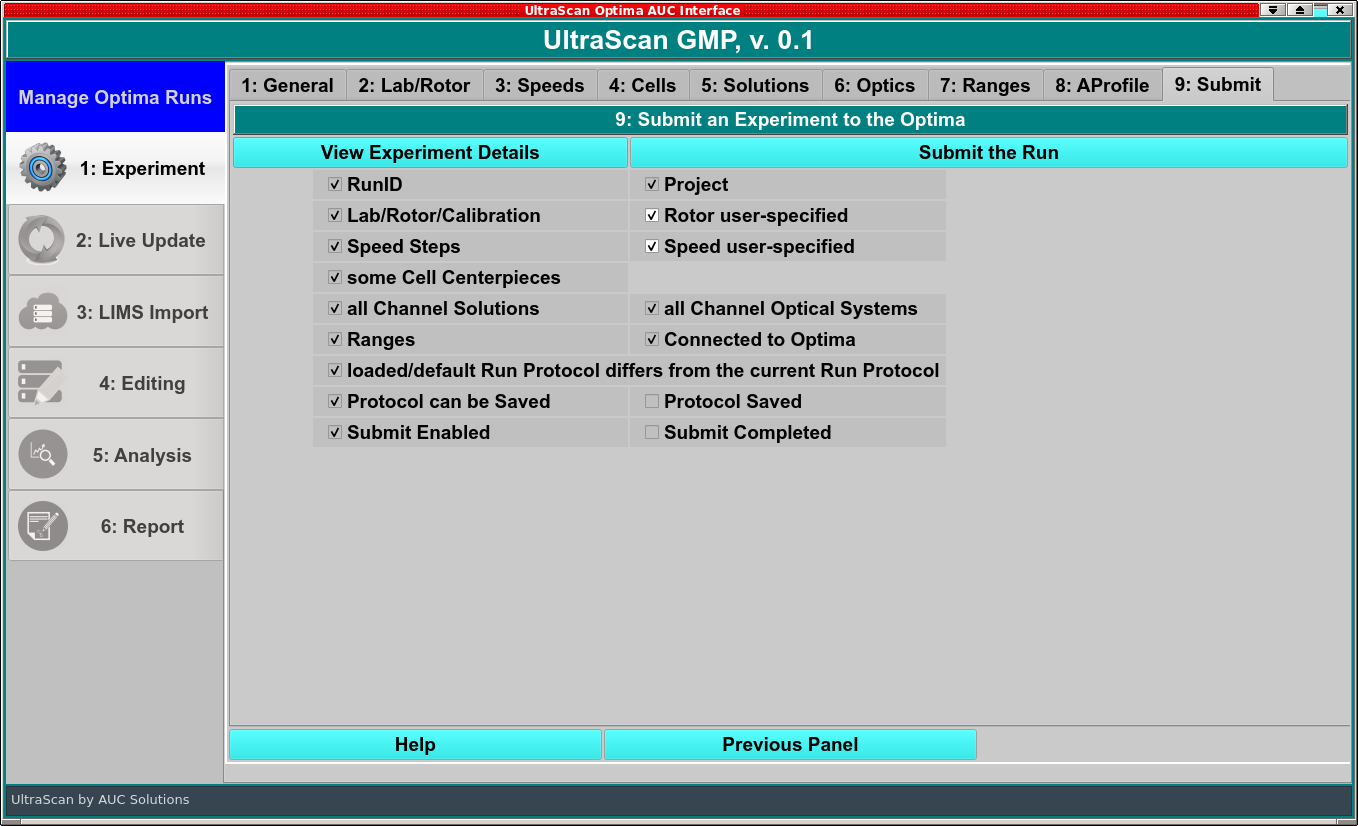

Supplement: S13 Fig — Submitting the run will save the profile in the UltraScan LIMS database in read-only mode, and send parameters required for executing data acquisition to the Optima instrument. (TIF) [file pcbi.1007942.s013.tif]

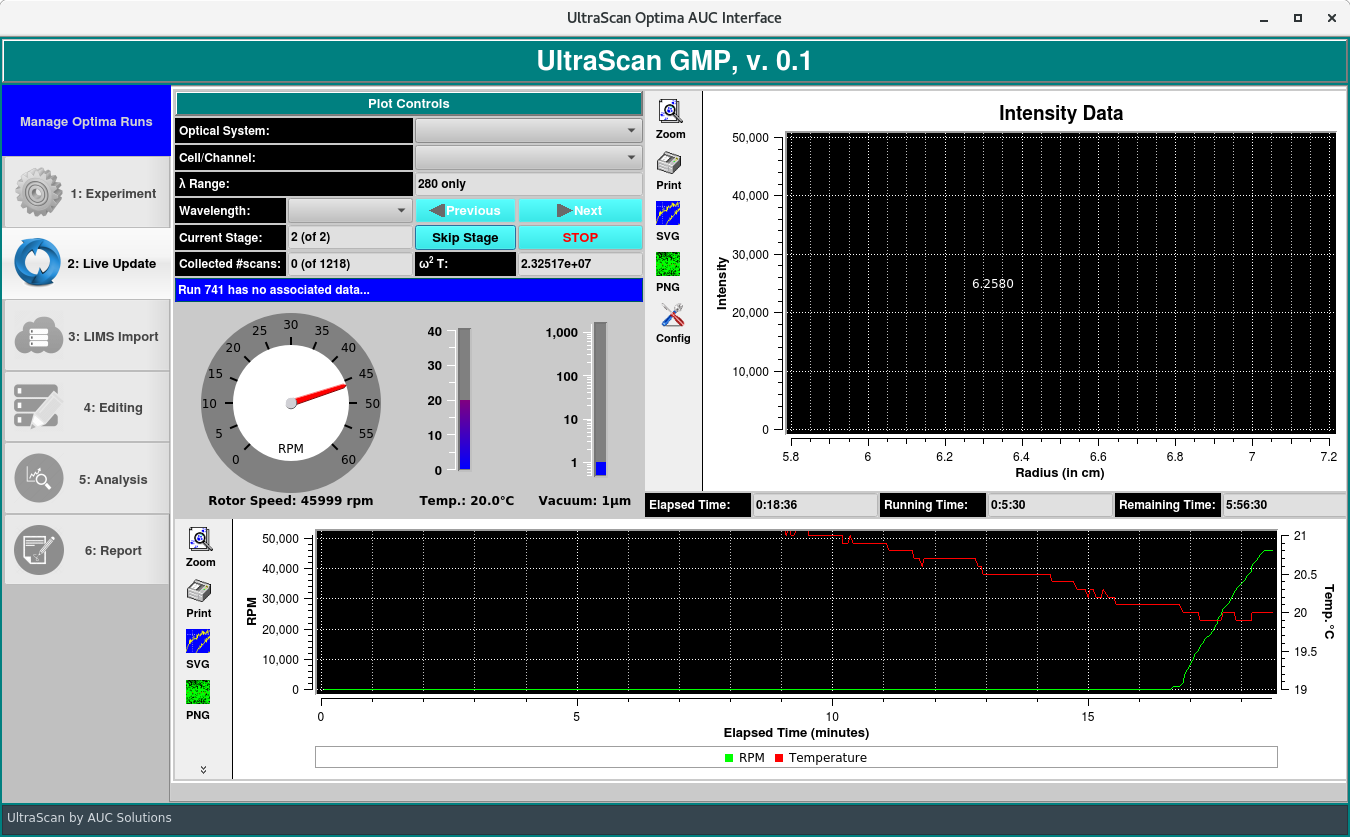

Supplement: S14 Fig — Science modules are being calibrated, and data acquisition has not yet started. Temperature and rotor speed are being monitored as soon as the run has been started on the instrument. (TIF) [file pcbi.1007942.s014.tif]

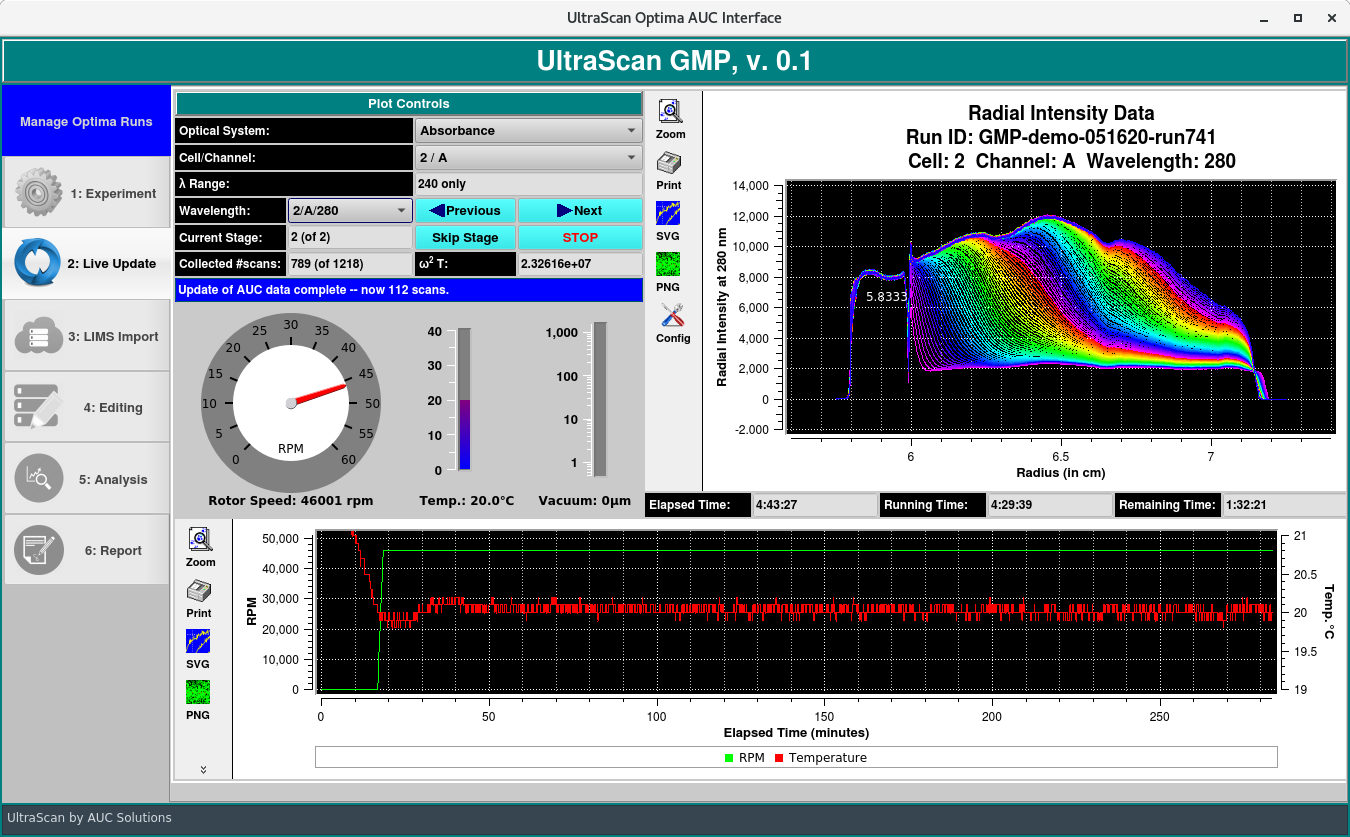

Supplement: S15 Fig — (TIF) [file pcbi.1007942.s015.tif]

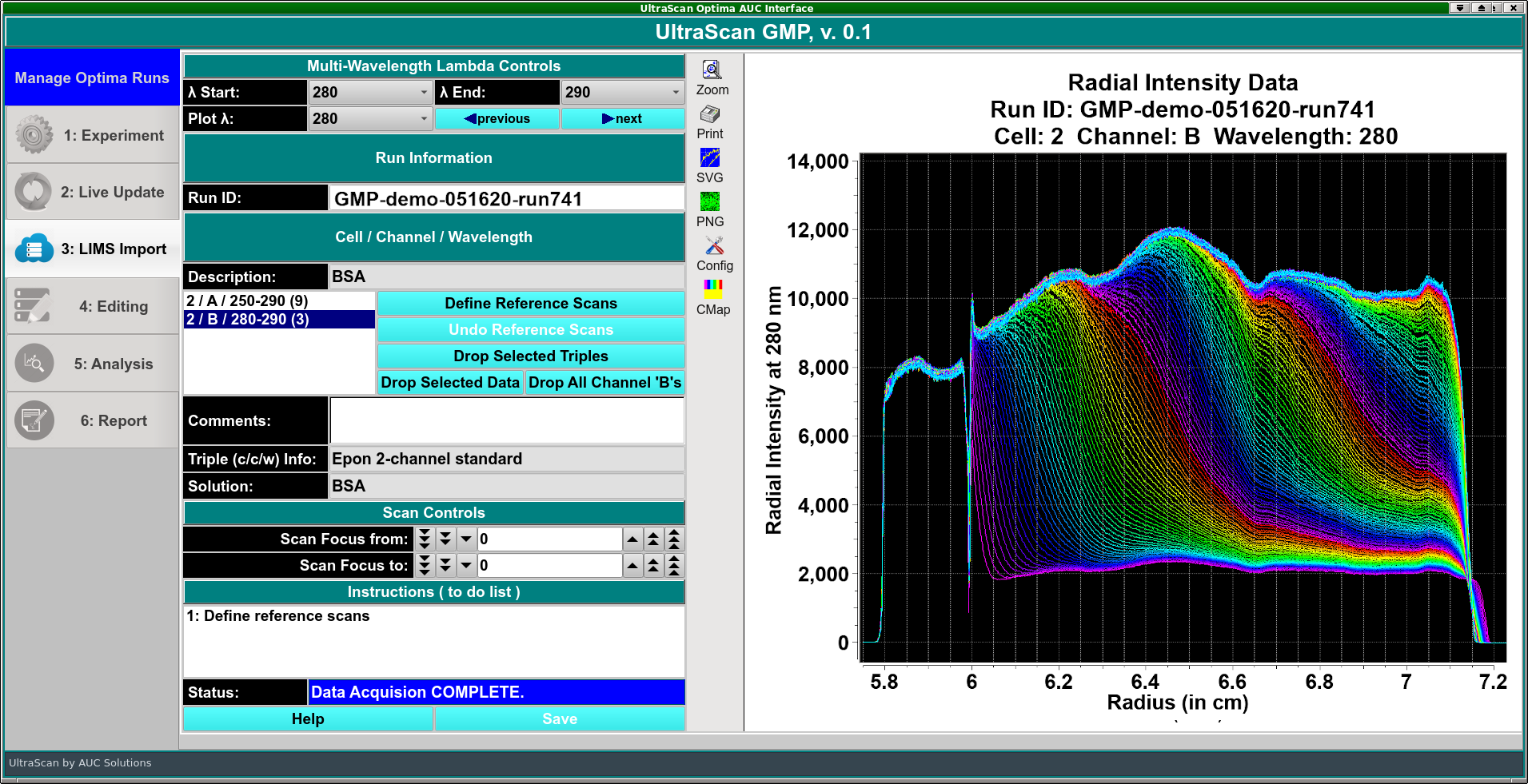

Supplement: S16 Fig — (TIF) [file pcbi.1007942.s016.tif]

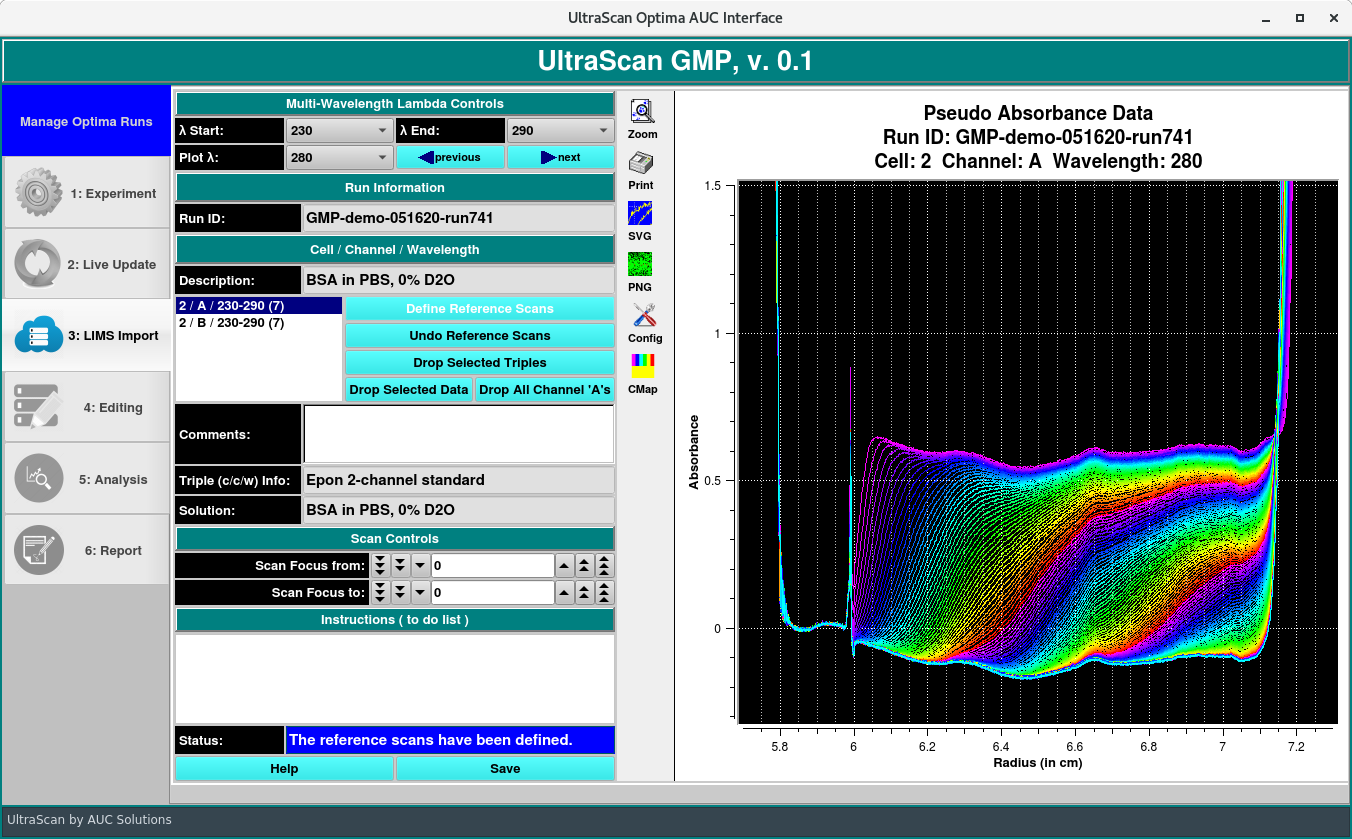

Supplement: S17 Fig — (TIF) [file pcbi.1007942.s017.tif]

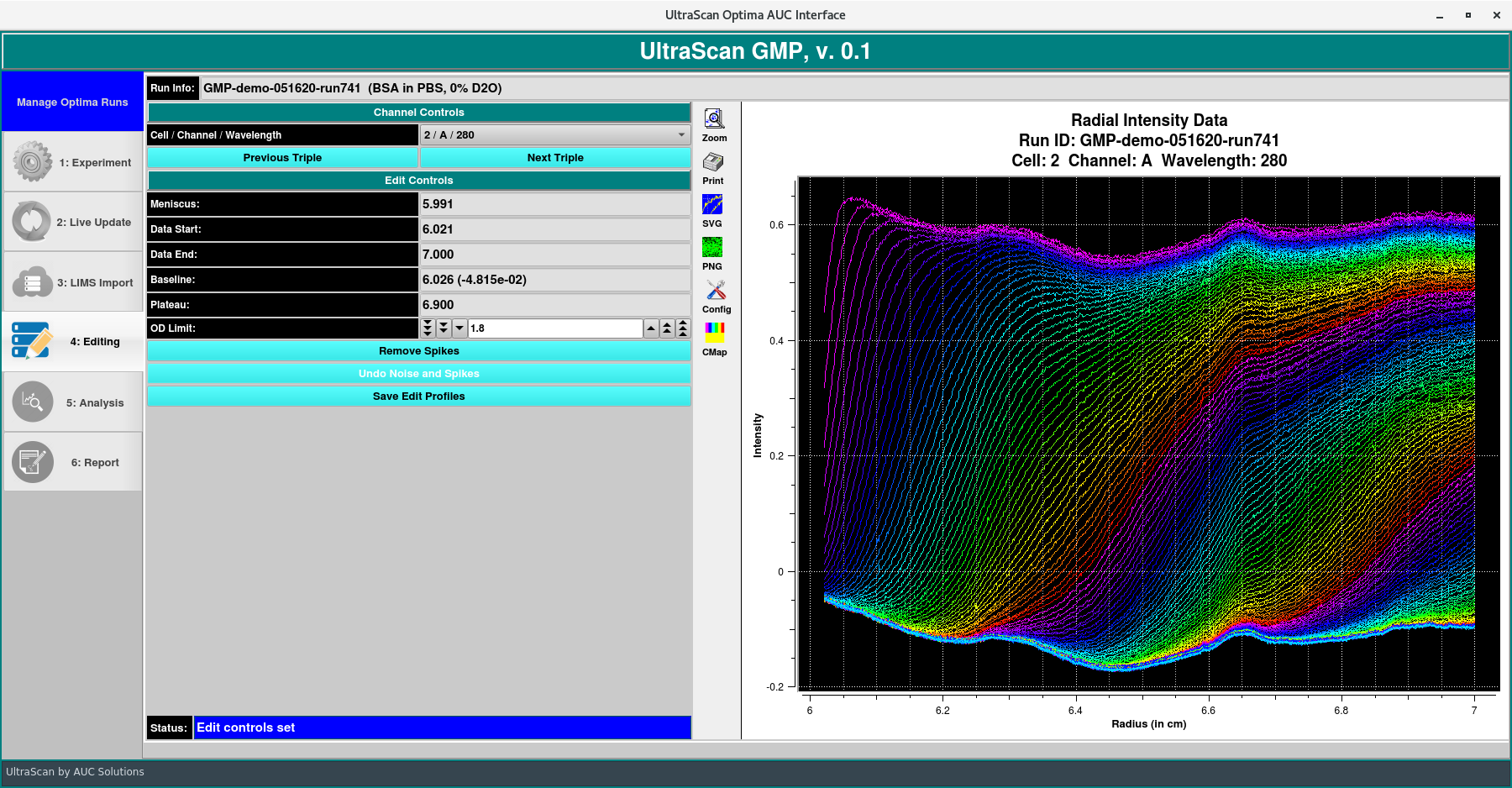

Supplement: S18 Fig — (TIF) [file pcbi.1007942.s018.tif]

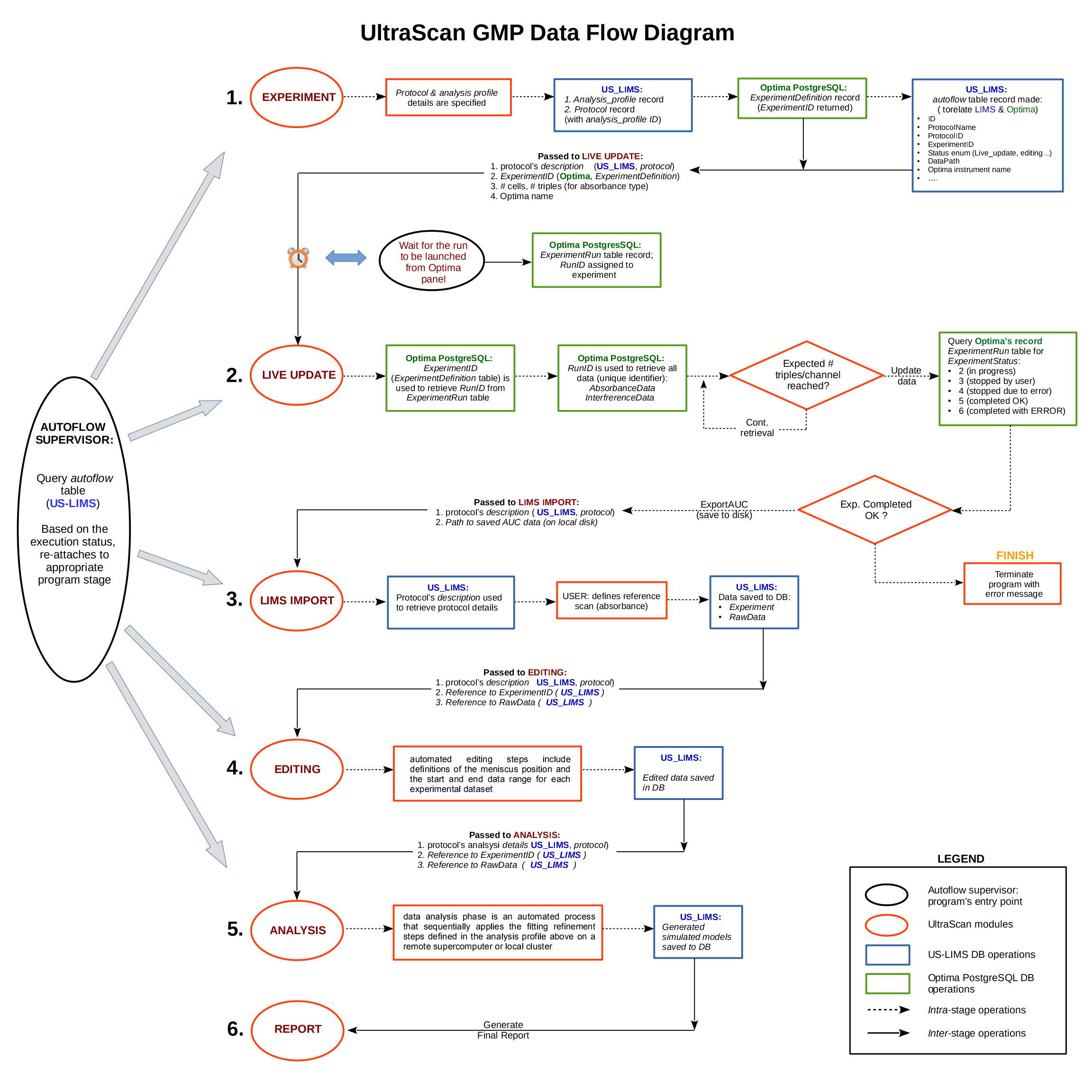

Supplement: S19 Fig — (TIF) [file pcbi.1007942.s019.tif]

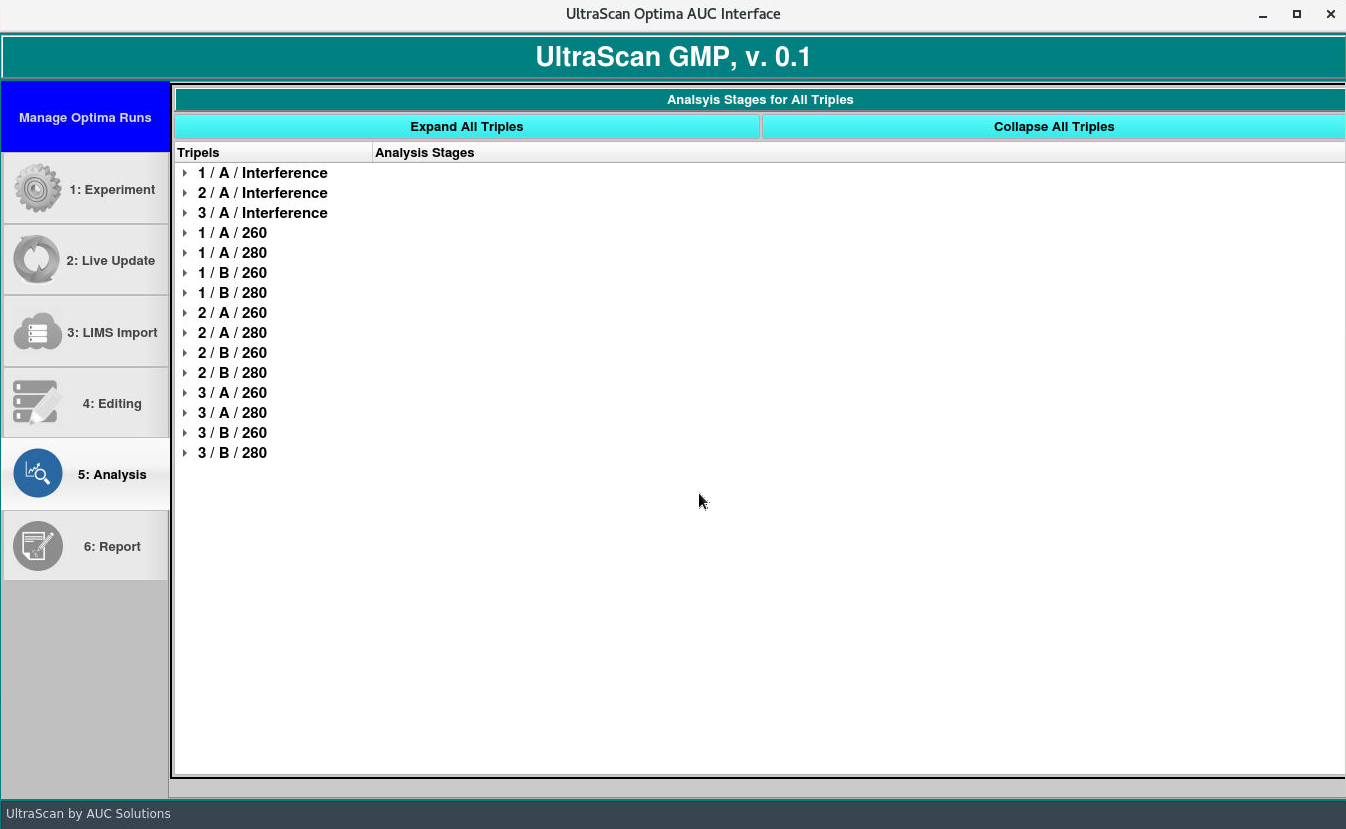

Supplement: S20 Fig — (TIF) [file pcbi.1007942.s020.tif]

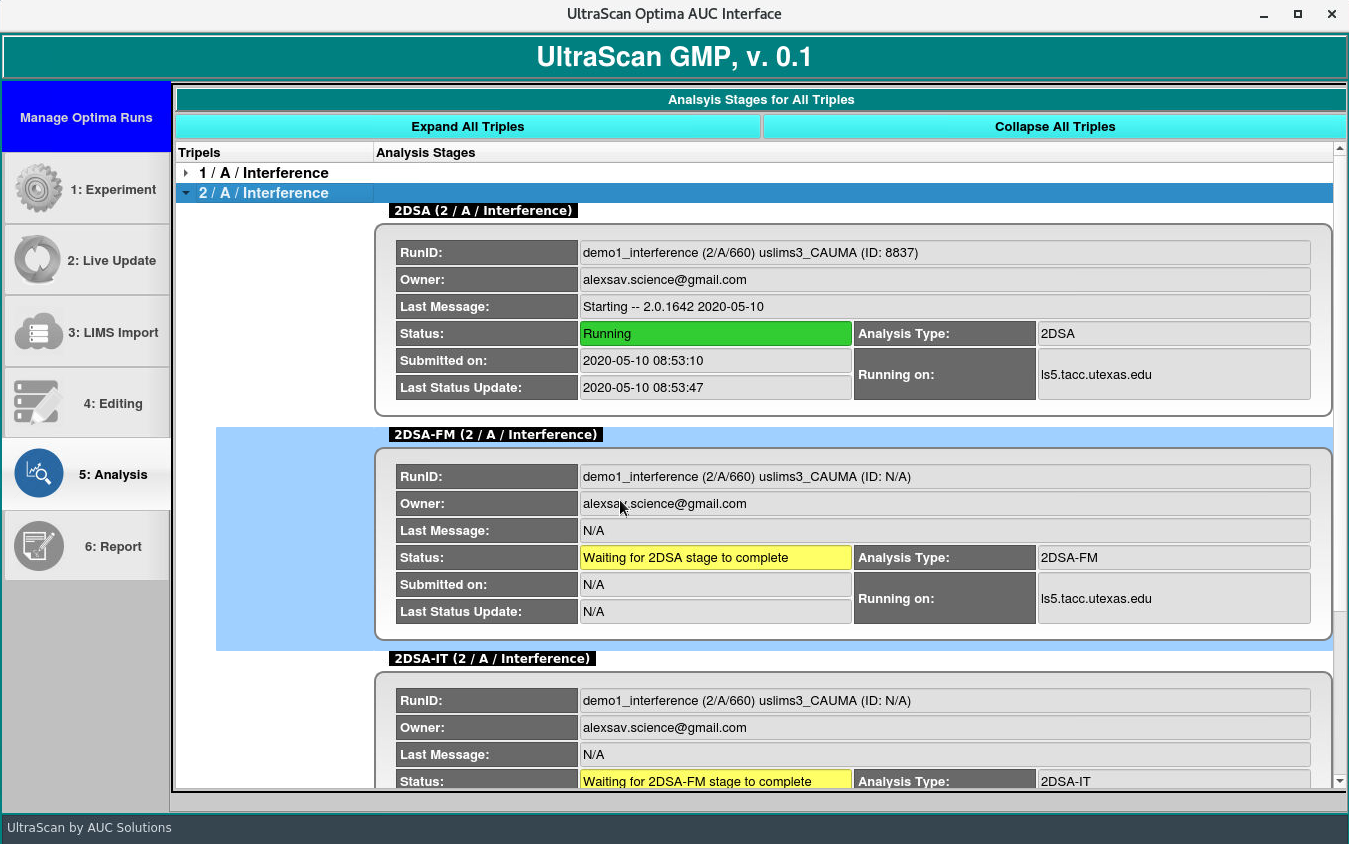

Supplement: S21 Fig — (TIF) [file pcbi.1007942.s021.tif]

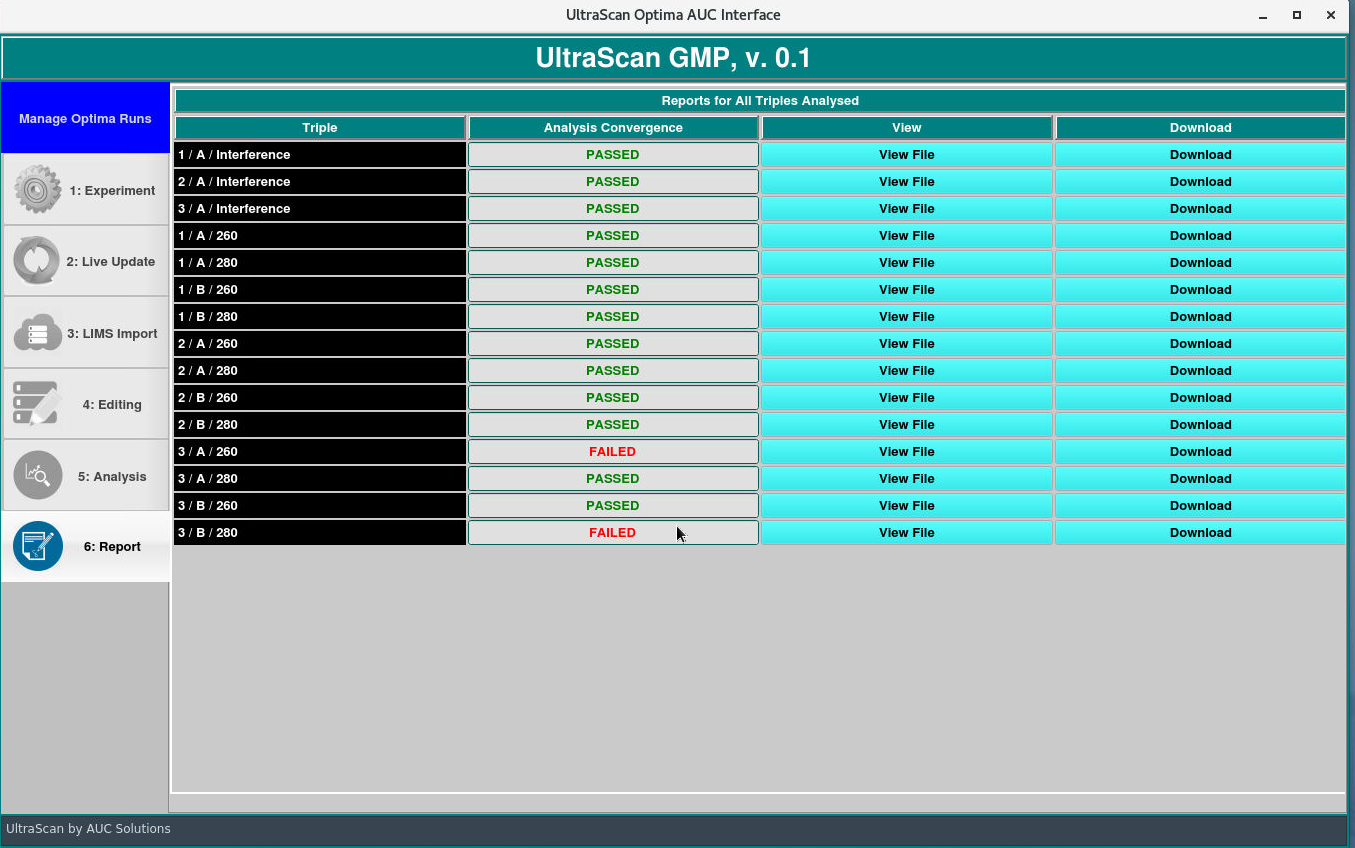

Supplement: S22 Fig — (TIF) [file pcbi.1007942.s022.tif]
